# Probable Hypocalcemia Induced Ventricular Fibrillation and Torsades de Pointes following Blood Product Administration

**DOI:** 10.7759/cureus.3765

**Published:** 2018-12-21

**Authors:** Christian M Mosebach, Jeffrey Kluger

**Affiliations:** 1 Internal Medicine, University of Connecticut, Farmington, USA; 2 Cardiology, Hartford Hospital, Hartford, USA

**Keywords:** hypocalcemia, torsades de pointes, blood transfusion, citrate, arrhythmia, ventricular arrhythmias

## Abstract

A 35-year-old male underwent open-heart surgery and required multiple blood product transfusions. Citrate, a preservative in blood products, caused serum ionized calcium chelation leading to hypocalcemia, a prolonged corrected QT (QTc) interval, and separate episodes of ventricular fibrillation and torsades de pointes (TdP). This case highlights an uncommon complication of blood product transfusion-induced hypocalcemia with precipitant arrhythmia.

## Introduction

Torsades de pointes (TdP) is a polymorphic ventricular tachycardia, which is characterized by twisting of the QRS axis around the points of the isoelectric line. An underrepresented cause of corrected QT (QTc) interval prolongation leading to TdP is hypocalcemia due to blood product transfusion. A recent study has shown that there is a statistically significant drop in serum ionized calcium after receiving just one unit of blood and that the severity of hypocalcemia increases with subsequent transfusions [1]. There are documented cases of blood product transfusions causing hypocalcemia and separate cases of hypocalcemia precipitating arrhythmias such as TdP or ventricular fibrillation. However, after an extensive literature review, we have found that there are no probable or definitive case reports of hypocalcemia induced arrhythmias such as TdP or ventricular fibrillation following blood product administration [1-3].

## Case presentation

**Figure 1:**
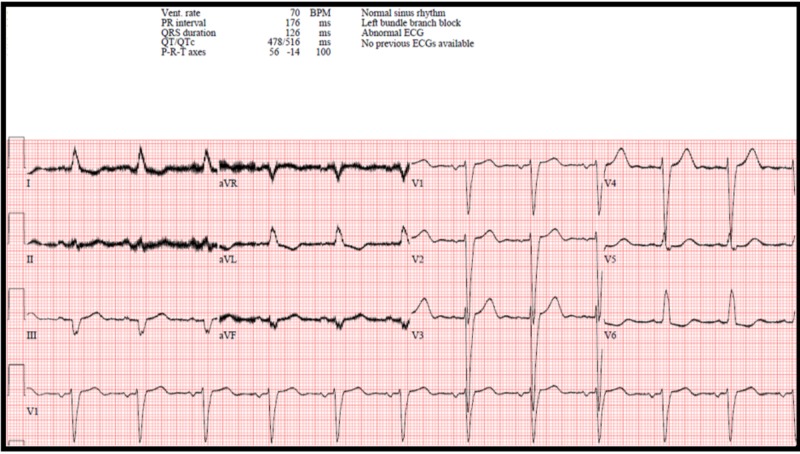
Admission electrocardiogram Electrocardiogram (ECG) showing the prolonged corrected QT (QTc) interval of 516 ms, which was further corrected to 469 ms to account for his left bundle branch block [4]. The ventricular (vent.) rate was 70 beats per minute (BPM).

A 35-year-old male, with no family history of cardiovascular disease or early unexpected cardiovascular death, presented to the hospital with bilateral leg swelling, shortness of breath, and chest heaviness. Review of his surgical history revealed a recent aortic root and aortic valve replacement due to infective endocarditis. On admission an electrocardiogram (ECG) showed a recalculated QTc of 469 ms when accounting for his left bundle branch block (LBBB) (Figure 1) [4]. Pertinent admission labs included aspartate aminotransferase (AST) 150 U/L (10–55 U/L), alanine aminotransferase (ALT) 119 U/L (10–55 U/L), creatinine 1.3 mg/dl (0.5–1.3 mg/dl), and blood urea nitrogen (BUN) 15 mg/dL (8–21 mg/dL). An echocardiogram was performed and showed a dehisced aortic valve which required urgent surgical repair. His operation was complicated by blood loss and coagulopathy, which required the transfusion of two units of packed red blood cells, two units of cryoprecipitate, four units of fresh frozen plasma, and two units of platelets. The patient had an intraoperative episode of hypocalcemia with a drop in serum ionized calcium from 1.20 mmol/L to 0.91 mmol/L (1.17–1.33 mmol/L) following blood product transfusion, which then precipitated an episode of intraoperative ventricular fibrillation. During this time, the patient's serum potassium was 3.6 mmol/L (3.4–5.3 mmol/L) and his serum magnesium was 2.1 mg/dl (1.6–2.7 mg/dl).

**Figure 2:**
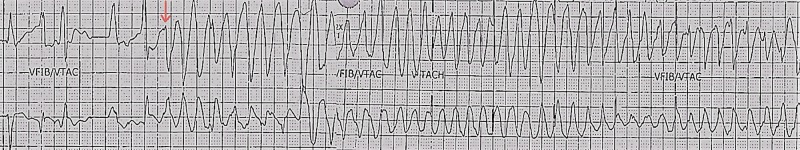
Telemetry strip showing torsades de pointes The red arrow in the figure above shows the onset of torsades de pointes. Note the telemetry interpretation is ventricular fibrillation/ventricular tachycardia (VFIB/VTAC).

During the post-operative period, the patient received an additional three units of packed red blood cells and one unit of platelets. The patient then had another drop in his serum ionized calcium from 1.32 mmol/L to 1.10 mmol/L, which precipitated an episode of TdP (Figure 2). His serum electrolytes at this time were potassium 3.4 mmol/L and magnesium 2.1 mg/dl. There were no new medications administered prior to this event, except for the administration of blood products containing the preservative citrate. Pertinent post-operative labs included AST 171 U/L (10–55 U/L), ALT 72 U/L (10–55 U/L), creatinine 1.2 mg/dl (0.5–1.3 mg/dl), and BUN 15 mg/dL (8–21 mg/dL).

**Figure 3:**
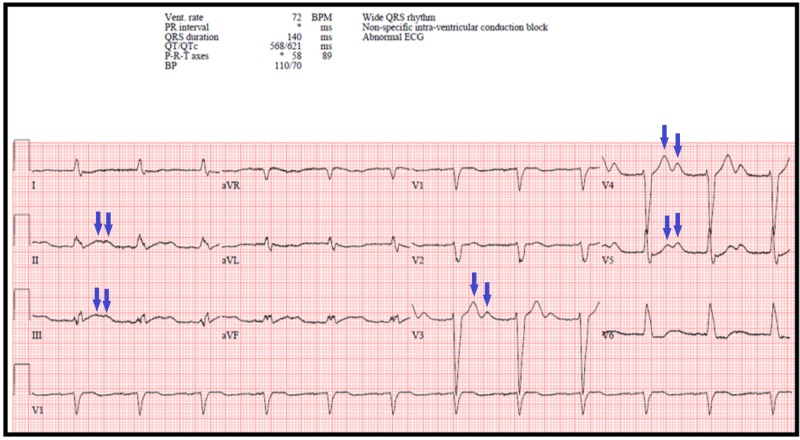
Electrocardiogram obtained directly after the episode of torsades de pointes Electrocardiogram (ECG) showing QTc of 562 ms when accounting for his wide QRS rhythm [4]. Bifid T-waves in leads II, III, V3-V5 are present (blue arrows), which is a characteristic of an acquired prolonged corrected QT (QTc) interval. The ventricular (vent.) rate was 72 beats per minute (BPM) and the blood pressure (BP) was 110/70.

**Figure 4:**
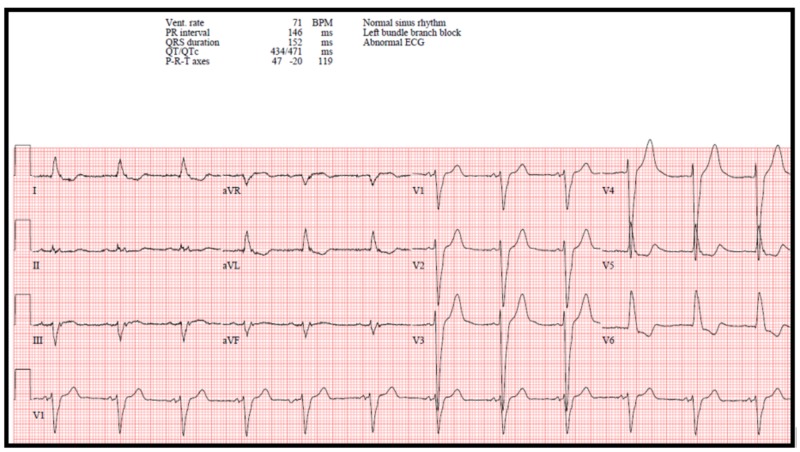
Electrocardiogram prior to discharge Electrocardiogram (ECG) with a left bundle branch block and corrected QT (QTc) interval of 401 ms when accounting for the wide QRS rhythm [4]. The bifid T-waves have now resolved with improvement in the patient's serum ionized calcium and normalization of the QTc interval. The ventricular (vent.) rate was 71 beats per minute (BPM).

Fortunately, the patient was defibrillated out of TdP within two minutes of going into the adventitious rhythm. An ECG performed immediately after the episode of TdP showed a prolonged QTc of 562 ms (Figure 3). Prior to discharge, and at least five days after the patient's last blood transfusion, he had an ECG which showed a significant improvement in his QTc to 401 ms (Figure 4). At the time of this ECG, his serum potassium was 3.5 mmol/L, magnesium 1.6 mg/dl, and ionized calcium 1.25 mmol/L. The patient continued to improve clinically and was subsequently discharged from the hospital with no further arrhythmias.

## Discussion

Throughout the patient's hospitalization, he received methadone 40 mg daily and ondansetron 4 mg IV approximately every 12 hours. Both of these medications are known to prolong the QTc and increase the risk of TdP [5]. Despite these medications contributing to the patient's prolonged QTc, he did not have any episodes of ventricular fibrillation or TdP until his serum ionized calcium levels had abruptly decreased after the administration of blood products containing citrate. The patient's serum potassium during both arrhythmias was between 3.4–3.6 mmol/L and his magnesium was 2.1 mg/dl in both cases. Based off of this data alone it could be hypothesized that his low normal potassium level contributed to the prolongation of his QTc; however, the ECG that was performed prior to discharge with a QTc of 401 ms was done when the patient's potassium was only 3.5 mmol/L. 

Hypocalcemia following blood product transfusion has been attributed to the interaction between serum ionized calcium and the preservative citrate [6]. In individuals with normal liver function, citrate is metabolized relatively quickly. Patients with underlying liver disease metabolize citrate at a slower rate leading to citrate toxicity and hypocalcemia [1,6]. The patient in this case was known to have a history of extensive polysubstance abuse and underlying liver disease with a baseline elevation of his AST and ALT. During the post-operative period he did not develop acute renal or liver injury or failure. His baseline liver dysfunction likely prolonged citrate metabolism and increased the chelation of ionized calcium resulting in hypocalcemia. 

Pathophysiology of hypocalcemia induced arrhythmias

**Figure 5:**
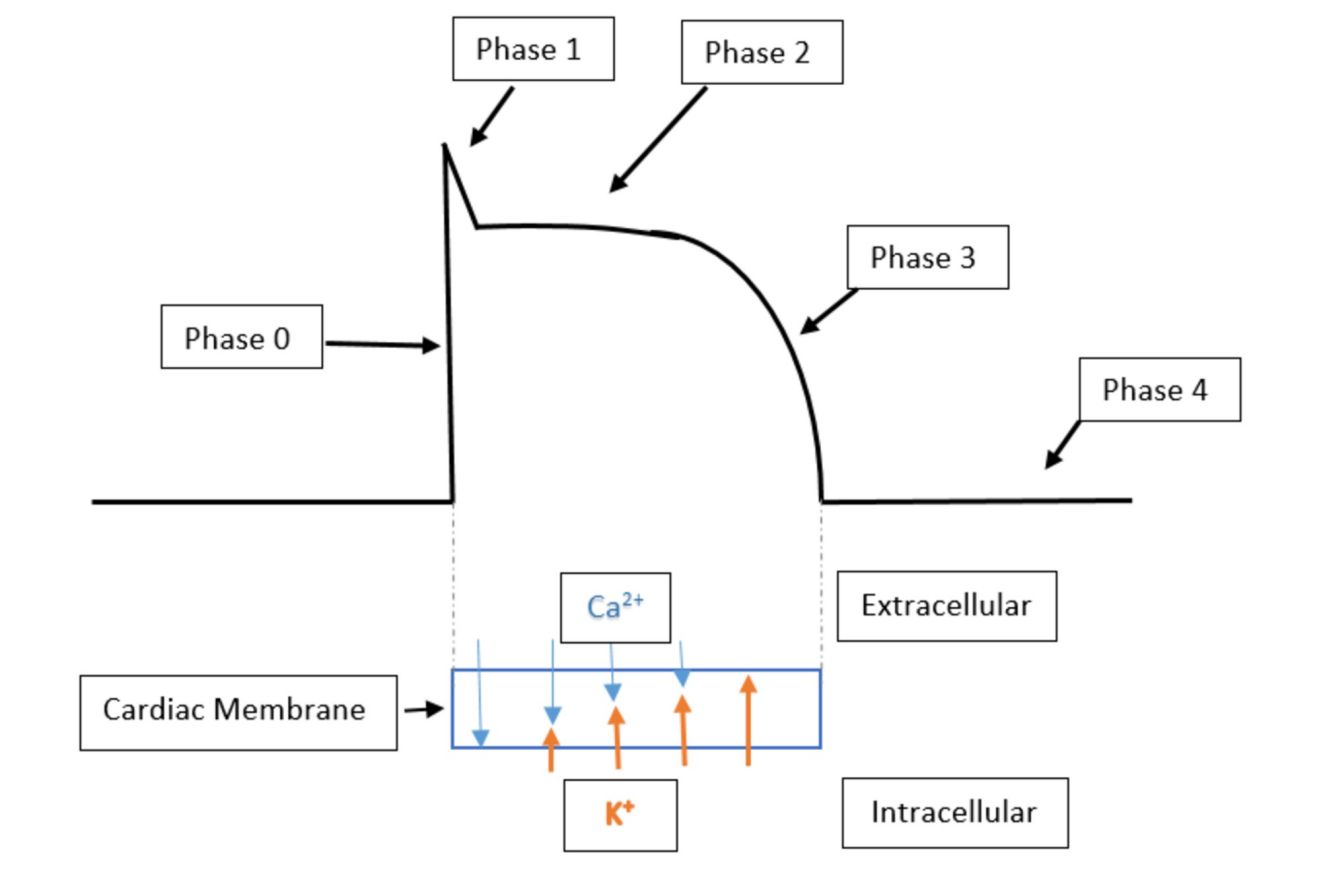
Normal ventricular action potential This image shows a normal ventricular action potential with phase 2 consisting of the calcium influx and potassium efflux. The balance of potassium and calcium flow during phase 2 gives the classic plateau shape to the ventricular action potential. Below the ventricular action potential is the relative flow of calcium (Ca2+) and potassium (K+) corresponding to their flow rate throughout phase 2 and 3.

**Figure 6:**
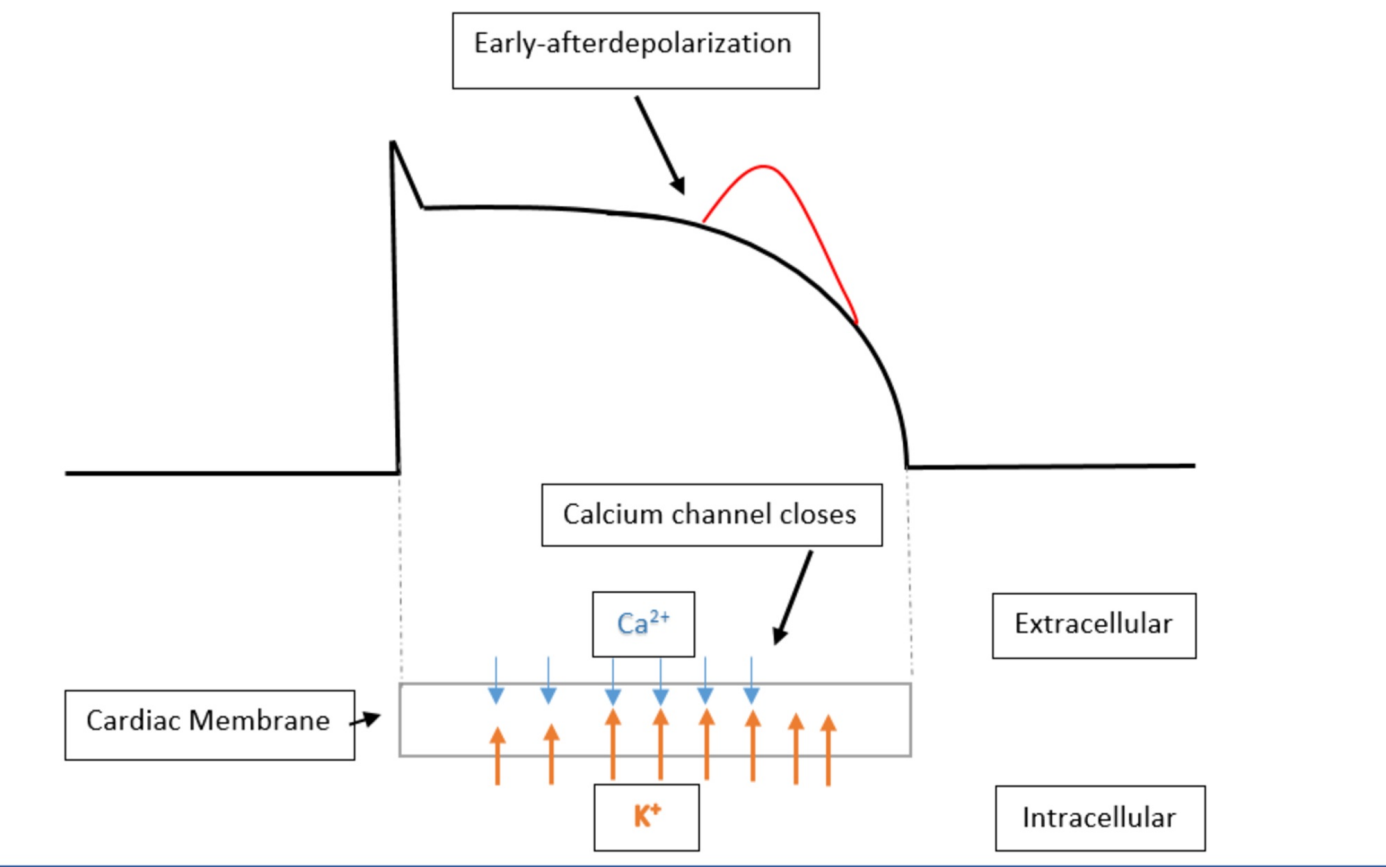
Abnormal ventricular action potential due to hypocalcemia induced QTc prolongation This figure shows the prolonged phase 2 and 3 of the ventricular action potential due to hypocalcemia. The lower half of this figure details the relative flow rates of calcium (Ca2+) and potassium (K+) through the cardiac membrane. When there is less free ionized calcium in systemic circulation, the rate of influx of calcium through the cardiac membrane and into the cardiac myocytes occurs at a slower rate. The closure of the cardiac myocyte calcium channels is thought to be regulated by total intracellular calcium concentration. The slower rate of calcium influx causes the calcium channels to be open for a longer period of time causing a prolongation of ventricular repolarization and subsequently a prolongation of the QTc interval. The red bump in the figure above indicates an early afterdepolarization occurring towards the end of phase 2. The propagation of an early afterdepolarization prior to the ventricular action potential reaching its baseline resting membrane potential is thought to be one of the ways in which an individual with a prolonged QTc could trigger an episode of torsades de pointes [7-8].

As the patient's serum ionized calcium levels decreased, there was likely a large intracellular shift of serum free ionized calcium. This intracellular calcium shift could be thought of as a protective mechanism as it is the intracellular calcium level that regulates the ventricular action potential length and therefore determines the length of the QTc interval [7]. There are many proposed mechanisms that have explained the trigger that closes the calcium channels involved in the phase 2 and 3 of the ventricular myocyte action potential. One mechanism shows that ventricular myocyte calcium channels will only close after the intracellular calcium concentration has increased to a specified threshold concentration [7]. If there is an abrupt decrease in the available ionized calcium, then the rate of intracellular calcium accumulation is slowed, which then prolongs the time that the calcium channel must remain open. If the calcium channel remains open for an extended period of time, it will cause a significant lengthening in the phase 2 and 3 of the myocardial action potential and therefore cause profound QTc lengthening (Figures 5-6). The QTc prolongation associated with transient hypocalcemia can then allow for an early afterdepolarization to precipitate TdP or ventricular fibrillation [8]. An early afterdepolarization is defined as a slowing or reversal of the normal repolarization during phase 2 or phase 3 of the ventricular action potential [8].

Causality analysis

An adverse drug reaction is defined as an appreciably harmful or unpleasant reaction, resulting from an intervention related to the use of a medicinal product, which predicts hazard from future administration and warrants prevention or specific treatment, or alteration of the dosage regimen, or withdrawal of the product [9]. A causality assessment tool can be used to evaluate the relationship between the administration of a particular treatment and the occurrence of an adverse reaction. There are a plethora of causality assessment tools that have been documented in the literature including the Naranjo algorithm, Yale algorithm, Jones algorithm, Karch algorithm, and the World Health Organization-Uppsala Monitoring Center (WHO-UMC) criteria [10-11]. Although there is no gold standard assessment tool, the most widely used decision aid is the Naranjo algorithm [11].

Using the Naranjo algorithm, we investigated if blood product administration containing citrate could cause hypocalcemia induced arrhythmias such as ventricular fibrillation or TdP? The “drug” that we used in our analysis was citrate, which was a preservative in the administered blood products that is known to cause hypocalcemia (Table 1) [6]. The patient received zero points for question number one as there are no previous conclusive reports on this reaction. He received +2 points for question number two and four as he did have an episode of ventricular fibrillation and then a separate episode of TdP with associated hypocalcemia after receiving the blood products containing citrate. The patient received +1 point for question number three as he did not have any episodes of hypocalcemia or cardiac arrhythmias when he was not receiving blood products containing citrate. We were generous and subtracted -1 point for question number five as the two episodes of arrhythmias could have been contributed to other QTc prolonging medications that he received; however, he was on these same medications (methadone and ondansetron) throughout the hospitalization and only had his two arrhythmias in the hours after receiving citrate containing blood products. The patient scored zero points for questions number six and seven as we did not administer a placebo and we did not attempt to measure the level of citrate in the patient's blood. The patient received +1 point for question number eight as he did have a more severe drop in his serum ionized calcium when he received a larger volume of blood products containing citrate. He received +1 point for both questions number nine and ten as he had two separate episodes of receiving citrate containing blood products with subsequent hypocalcemia induced arrhythmias. Per the Naranjo Adverse Drug Reaction Probability Scale, a score of nine or higher is defined as a "definite" adverse reaction, a score from five to eight is defined as a “probable” adverse reaction, and a score from one to four is defined as a "possible" adverse reaction [10]. Our case scores a seven, which indicates there was a probable relationship between blood product administration containing citrate and the development of hypocalcemia induced arrhythmias such as ventricular fibrillation or TdP (Table 1).

**Table 1:** Naranjo Adverse Drug Reaction Probability Scale Completed Naranjo scale for our case with a score of seven, which indicates a probable relationship between blood product administration containing citrate and the development of hypocalcemia induced arrhythmias such as ventricular fibrillation or torsades de pointes [10].

Question	Yes	No	Do Not Know		Score
1. Are there previous conclusive reports on this reaction?	+1	0	0		0
2. Did the adverse event appear after the suspected drug was administered?	+2	-1	0		+2
3. Did the adverse reaction improve when the drug was discontinued or a specific antagonist was administered?	+1	0	0		+1
4. Did the adverse event reappear when the drug was re-administered?	+2	-1	0		+2
5. Are there alternative causes (other than the drug) that could on their own have caused the reaction?	-1	+2	0		-1
6. Did the reaction reappear when a placebo was given?	-1	+1	0		0
7. Was the drug detected in the blood (or other fluids) in concentrations known to be toxic?	+1	0	0		0
8. Was the reaction more severe when the dose was increased or less severe when the dose was decreased?	+1	0	0		+1
9. Did the patient have a similar reaction to the same or similar drugs in any previous exposure?	+1	0	0		+1
10. Was the adverse event confirmed by objective evidence?	+1	0	0		+1
	Total Score				+7

## Conclusions

An underrepresented complication of blood product administration is hypocalcemia due to the preservative citrate binding to free serum ionized calcium. In patients requiring blood product administration, it is imperative to closely monitor serum ionized calcium and aggressively replace this vital electrolyte to reduce the risk of precipitating arrhythmias. This is the first known published case, to our knowledge, that has used a causality assessment tool to document the probable relationship between blood product transfusion-induced hypocalcemia and the development of arrhythmias such as ventricular fibrillation or TdP.

## References

[1] Webster S, Todd S, Redhead J, Wright C (2016). Ionised calcium levels in major trauma patients who received blood in the emergency department. Emerg Med J.

[2] Akiyama T, Batchelder J, Worsman J, Moses HW, Jedlinski M (1989). Hypocalcemic torsades de pointes. J Electrocardiol.

[3] Eryol N, Colak R, Ozdogru B (2003). Effects of calcium treatment on QT interval and QT dispersion in hypocalcemia. Am J Cardiol.

[4] Wang B, Zhang Li, Cong P (2017). A new formula for estimating the true QT interval in left bundle branch block. J Cardiovasc Electrophysiol.

[5] (2018). CredibleMeds. https://crediblemeds.org/index.php/drugsearch.

[6] Monchi M (2017). Citrate pathophysiology and metabolism. Transfus Apher Sci.

[7] Grandi E, Pasqualini FS, Pes C, Corsi C, Zaza A, Severi S (2009). Theoretical investigation of action potential duration dependence on extracellular Ca2+ in human cardiomyocytes. J Mol Cell Cardiol.

[8] Weiss JN, Garfinkel A, Karagueuzian HS, Chen PS, Qu Z (2010). Early afterdepolarizations and cardiac arrhythmias. Heart Rhythm.

[9] Edwards IR, Aronson JK (2000). Adverse drug reactions: definitions, diagnosis, and management. Lancet.

[10] Naranjo CA, Busto U, Sellers EM (1981). A method for estimating the probability of adverse drug reactions. Clin Pharmacol Ther.

[11] Srinivasan R, Ramya G (2011). Adverse drug reaction - causality assessment. Int J Res Pharm Chem.

